# Crystal structure, Hirshfeld surface analysis and DFT studies of 2-[(2-hy­droxy-5-methyl­benzyl­idene)amino]­benzo­nitrile

**DOI:** 10.1107/S2056989020008907

**Published:** 2020-07-03

**Authors:** Md. Serajul Haque Faizi, Emine Berrin Cinar, Alev Sema Aydin, Erbil Agar, Necmi Dege, Ashraf Mashrai

**Affiliations:** a Department of Chemistry, Langat Singh College, B.R.A. Bihar University, Muzaffarpur, Bihar-842001, India; b Ondokuz Mayıs University, Faculty of Arts and Sciences, Department of Physics, Samsun, Turkey; c Ondokuz Mayıs University, Faculty of Arts and Sciences, Department of Chemistry, Samsun, Turkey; d Department of Pharmacy, University of Science and Technology, Ibb Branch, Ibb, Yemen

**Keywords:** crystal structure, 2-hy­droxy-5-methyl-benzaldehyde, 2 amino­benzo­nitrile, Schiff base

## Abstract

The title compound, C_15_H_12_N_2_O, was synthesized by condensation reaction of 2-hy­droxy-5-methyl­benzaldehyde and 2-amino­benzo­nitrile, and crystallizes in the ortho­rhom­bic space group *Pbca*. The phenol ring is inclined to the benzo­nitrile ring by 25.65 (3)°. The configuration about the C=N bond is *E*, stabilized by a strong intra­molecular O—H⋯N hydrogen bond that forms an *S*(6) ring motif. In the crystal, C—H⋯O and C—H⋯N inter­actions lead to the formation of sheets perpendicular to the *a* axis. C—H⋯π inter­actions, forming polymeric chains along the *a-*axis direction, connect these sheets into a three-dimensional network. A Hirshfeld surface analysis indicates that the most important contributions for the packing arrangement are from H⋯H and C⋯H/H⋯C inter­actions. The density functional theory (DFT) optimized structure at the B3LYP/6–311 G(d,p) level is compared with the experimentally determined mol­ecular structure and the HOMO–LUMO energy gap is given.

## Abstract 2

The title compound crystallizes in the ortho­rhom­bic space group *Pbca*. The phenol ring is inclined to the benzo­nitrile ring by 25.65 (3)°. The configuration about the C=N bond is *E*, stabilized by a strong intra­molecular O—H⋯N hydrogen bond that forms an *S*(6) ring motif.

## Chemical context   

Schiff bases containing the azomethine moiety (–*R*CH=N–*R*′) are prepared by a condensation reaction between amines and reactive carbonyl compounds, such as aldehydes. Schiff bases are employed as catalyst carriers (Grigoras *et al.*, 2001 ▸), thermo-stable materials (Vančo *et al.*, 2004 ▸), metal–cation complexing agents and in biological systems (Taggi *et al.*, 2002 ▸). They also show biological activities such as anti­bacterial, anti­fungal, anti­cancer, anti­viral and herbicidal (Desai *et al.*, 2001 ▸; Singh & Dash, 1988 ▸; Karia & Parsania, 1999 ▸; Siddiqui *et al.*, 2006 ▸). Schiff bases are also capable of forming stable complexes by coordination to metal ions *via* their nitro­gen donor atoms (Ebrahimipour *et al.*, 2012 ▸). They are important for their photochromic properties and have applications in various fields such as the measurement and control of radiation intensities in imaging systems and in optical computers, electronics, optoelectronics and photonics (Iwan *et al.*, 2007 ▸). *ortho*-Hy­droxy Schiff base compounds such as the title compound can display two tautomeric forms, the enol–imine (OH) and keto–amine (NH) forms. Depending on the tautomers, two types of intra­molecular hydrogen bonds are generally observed in *ortho*-hy­droxy Schiff bases, namely O—H⋯N in enol–imine and N—H⋯O in keto–amine tautomers (Tanak *et al.*, 2010 ▸). The present work is a part of an ongoing structural study of Schiff bases and their utilization in the synthesis of quinoxaline derivatives (Faizi *et al.*, 2018 ▸), fluorescence sensors (Faizi *et al.*, 2016 ▸ ▸; Mukherjee *et al.*, 2018 ▸; Kumar *et al.*, 2017 ▸; 2018 ▸) and non-linear optical properties (Faizi *et al.*, 2020 ▸). We report herein the synthesis of the title compound 2-[(2-hy­droxy-5-methyl­benzyl­idene)amino]­benzo­nitrile (I) from 2-hy­droxy-5-methyl­benzaldehyde and 3-chloro-4-methyl­aniline, as well as its crystal structure, Hirshfeld surface analysis and DFT computational calculations. The results of calculations by density functional theory (DFT) carried out at the B3LYP/6–311 G(d,p) level are compared with the experimentally determined mol­ecular structure in the solid state.
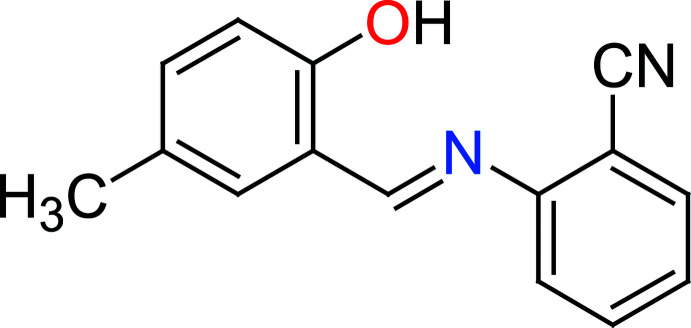


## Structural commentary   

**Figure 1:**
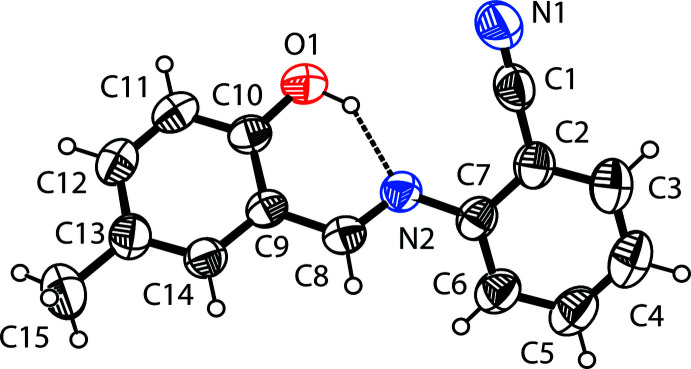
The mol­ecular structure of (I) with the atom-numbering scheme. Displacement ellipsoids are drawn at the 40% probability level. The intra­molecular O—H⋯N hydrogen bond (Table1) is shown as a dashed line.

The mol­ecular structure of the title compound is shown in Fig.1. The configuration of the C8=N2 bond of this Schiff base is *E*, stabilized by the intra­molecular O1—H1⋯N1 hydrogen bond that forms an *S*(6) ring motif (Fig. 1 ▸ and Table 1 ▸). This is a relatively common feature in analogous imine-phenol compounds (see *Database survey* section). The C10—O1 bond length [1.3503 (17) Å for X-ray and 1.337 Å for B3LYP] indicates single-bond character, while the imine C8=N2 bond length [1.2795 (17)Å for X-ray and 1.291 Å for B3LYP] indicates double-bond character. All bond lengths and bond angles are within normal ranges and are comparable with those in related Schiff base compounds (Faizi *et al.*, 2019 ▸; Kansiz *et al.*, 2018 ▸; Ozeryanskii *et al.*, 2006 ▸). The C10—O1 and C8=N2 bond lengths confirm the enol–imine form of the title compound (Wozniak *et al.*, 1995 ▸; Pizzala *et al.*, 2000 ▸). The mol­ecule is not planar, with the benzo­nitrile ring tilted by 25.65 (3)° to the plane of the 5-methyl­phenol moiety. The imine and 5-methyl­phenol groups are, however, essentially coplanar, as indicated by the C9—C8—N2—C7 torsion angle of −178.75 (13)° and the C1—C14—C15—N1 torsion angle [0.31 (3)° for X-ray and 0.44° for B3LYP].

## Supra­molecular features and Hirshfeld surface analysis   

**Figure 2:**
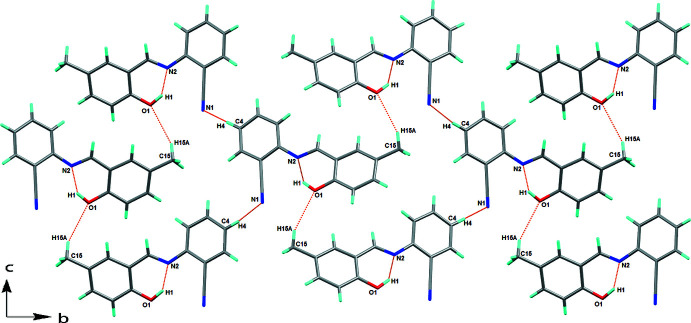
View along the *a* axis of the unit cell showing the mol­ecular sheets, formed *via* C—H⋯O and C—H⋯N inter­actions (see Table 1 ▸ for details).

**Figure 3:**
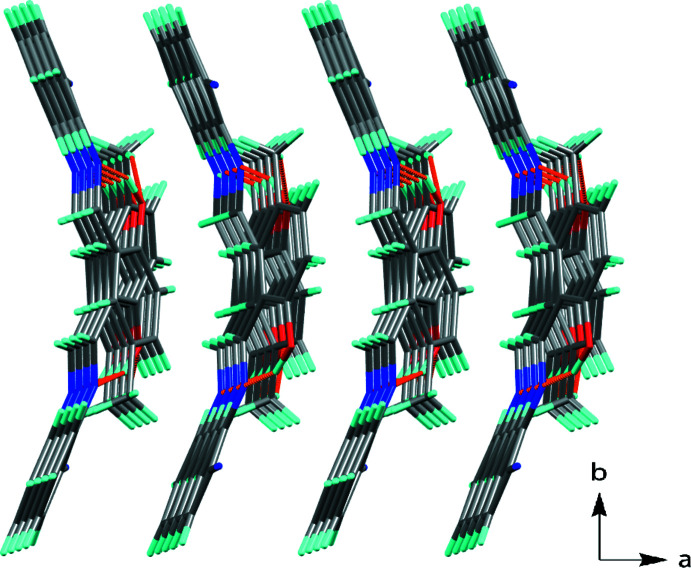
View along the *c* axis of the unit cell showing the infinite chains, formed *via* C—H⋯π inter­actions (see Table 1 ▸ for details).

The crystal structure of the title compound is consolidated by C—H⋯O and C—H⋯N inter­actions, forming corrugated layers perpendicular to the *a* axis (Fig. 2 ▸, Table 1 ▸). The mol­ecules are also linked through inter­molecular C—H⋯π inter­actions hydrogen atom H11 and the centroid of the C9–C14 ring at 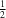 + *x*, 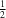 − *y*, 1 − *z*, which connect mol­ecules along the *a*-axis direction (Fig. 3 ▸). C—H⋯O, C—H⋯N and C—H⋯π inter­actions combined lead to the formation of a three-dimensional network.

**Figure 4:**
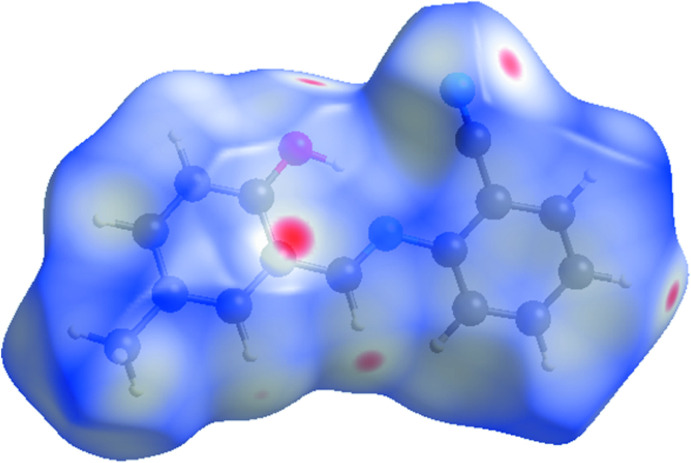
The Hirshfeld surface of compound (I) mapped over *d*_norm_.

**Figure 5:**
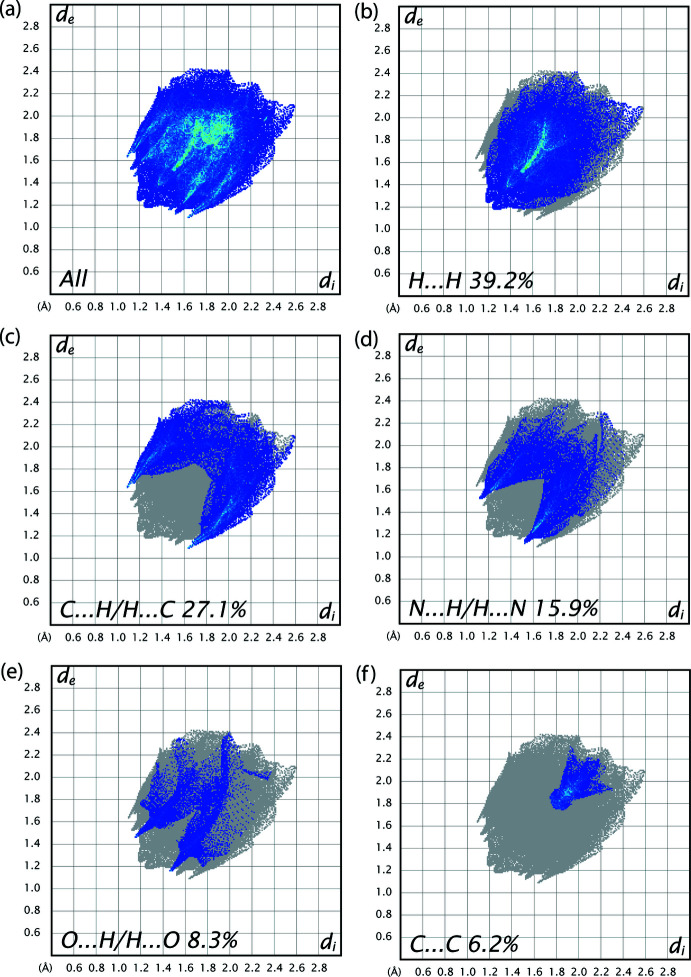
The overall two-dimensional finger print plots for compound (I), and those delineated into: (*b*) H⋯H (39.2%), (*c*) C⋯H/H⋯C (27.1%), (*d*) N⋯H/H⋯N (16.0%), (*e*) O⋯H/H⋯O (8.3%) and C⋯C (6.2%) contacts.

In order to better visualize and analyze the role of weak inter­molecular contacts in the crystal, a Hirshfeld surface (HS) analysis (Spackman & Jayatilaka, 2009 ▸) was carried out and the associated two-dimensional fingerprint plots (McKinnon *et al.*, 2007 ▸) generated using *CrystalExplorer17.5* (Turner *et al.*, 2017 ▸) were analysed. The three-dimensional *d*_norm_ surface is shown in Fig. 4 ▸ with a standard surface resolution and a fixed colour scale of −0.1805 to 1.0413 a.u. The darkest red spots on the Hirshfeld surface indicate contact points with atoms participating in the C—H⋯π inter­actions involving C11—H11 and the phenyl substituent (Table 1 ▸). As illustrated in Fig. 5 ▸ *a*, the corresponding fingerprint plots for the compound have characteristic pseudo-symmetric wings along the *d*_e_ and *d*_i_ diagonal axes. The presence of C—H⋯π inter­actions in the crystal is indicated by the pair of characteristic wings in the fingerprint plot delineated into C⋯H/H⋯C contacts (Fig. 5 ▸ *c*, 27.1% contribution to the Hirshfeld surface). As shown in Fig. 5 ▸ *b*, the most widely scattered points in the fingerprint plot are related to H⋯H contacts, which make a contribution of 39.2% to the Hirshfeld surface. There are also N⋯H/H⋯N (16.0%; Fig. 5 ▸ *d*), O⋯H/H⋯O (8.3%; Fig. 5 ▸ *e*) and C⋯C (6.2%; Fig. 5 ▸ *f*) contacts, with smaller contributions from C⋯N/N⋯C (2.6%), C⋯O/O⋯C (0.4%) and N⋯N (0.3%) contacts.

## DFT calculations   

The optimized structure of the title compound in the gas phase was generated theoretically *via* density functional theory (DFT) calculations using the standard B3LYP functional and a 6-311G(d,p) basis-set (Becke, 1993 ▸) as implemented in *GAUSSIAN09* (Frisch *et al.*, 2009 ▸). The theoretical and experimental results are in good agreement (Table 2 ▸). The C8=N2 bond length is 1.2795 (17) Å for X-ray and 1.291 Å for B3LYP and the C10—O1 bond length is 1.3503 (17) Å for X-ray and 1.367 Å for B3LYP.

The highest-occupied mol­ecular orbital (HOMO) and the lowest-unoccupied mol­ecular orbital (LUMO) are very important parameters for quantum chemistry. Many electronic, optical and chemical reactivity properties of compounds can be predicted from frontier mol­ecular orbitals (Tanak, 2019 ▸). A mol­ecule with a small HOMO–LUMO bandgap is more polarizable than one with a large gap and is considered a soft mol­ecule because of its high polarizibility, while mol­ecules with a large bandgap are considered to be ‘hard mol­ecules’. To better understand the nature of the title compound, the electron affinity (*A* = -*E*_HOMO_), the ionization potential (*I* = -*E*_LUMO_), HOMO–LUMO energy gap (Δ*E*), the chemical hardness (η) and softness (*S*) of the title compound were predicted based on the *E*_HOMO_ and *E*_LUMO_ energies (Tanak, 2019 ▸). For the title compound, *I* = 6.146 eV, *A* = 2.223 eV, Δ*E* = 3.923 eV, η = 1.961 eV and *S* = 0.311 eV. Based on the relatively large Δ*E* and η values, the title compound can be classified as a hard mol­ecule.

**Figure 6:**
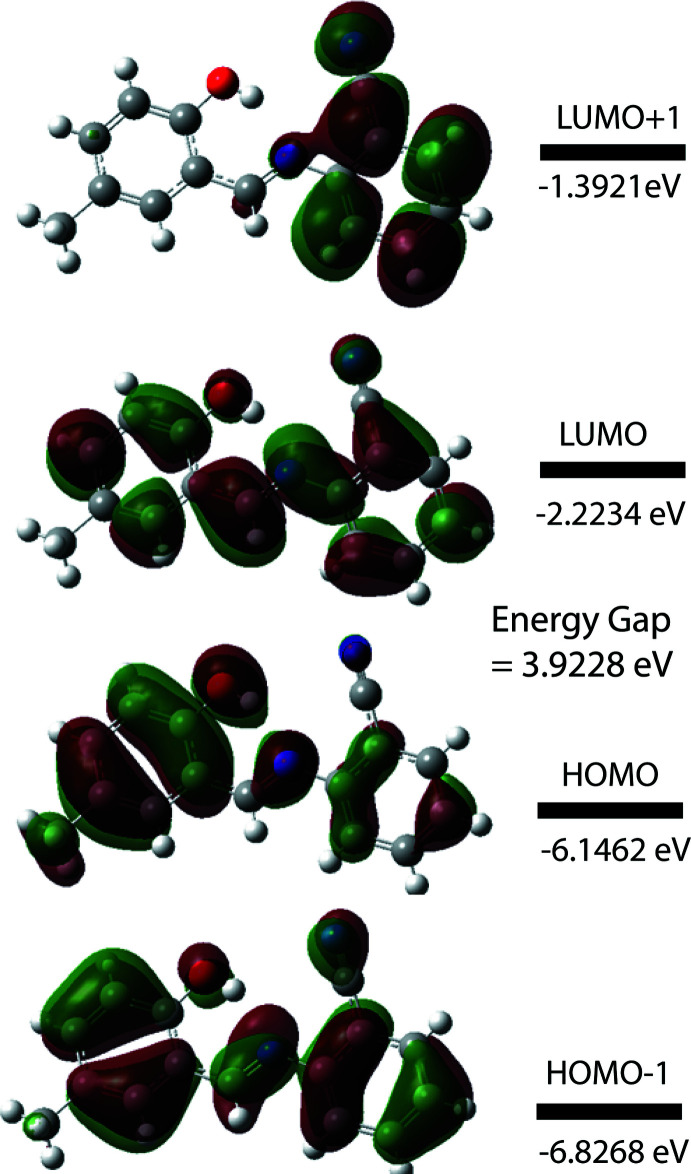
Electron distribution of the HOMO-1, HOMO, LUMO and the LUMO+1 energy levels for the title compound.

The electron distribution of the HOMO-1, HOMO, LUMO and the LUMO+1 energy levels are shown in Fig. 6 ▸. The DFT study shows that the HOMO and LUMO are localized in the plane extending from the whole 2-hy­droxy-5-methyl-benzaldehyde ring to the 2 amino­benzo­nitrile unit. The HOMO, HOMO-1 and LUMO orbitals are delocalized over the π systems of the two aromatic rings and connected by the Schiff base bridge. HOMO and HOMO-1 can be said to be π-bonding with respect to the C=N imine bond, while the LUMO orbital has imine π* anti­bonding character. The LUMO+1 orbital on the other hand is localized only on the amino­benzo­nitrile ring and the C atom of the Schiff base. With respect to the imine π-bond it is mostly non-bonding. From the frontier orbital analysis, it can be concluded that a HOMO-to-LUMO excitation of (I) would be a π–π* transition that would weaken the imine bond and drive the production of an excited-state keto–amine tautomer from the enol–imine ground state observed in the solid state. The calculated bandgap of (I) is 3.923 eV, which is similar to that reported for other Schiff base materials, such as for example (*E*)-2-{[(3-chloro­phen­yl)imino]­meth­yl}-6-methyl­phenol (energy gap = 4.069 eV; Faizi *et al.*, 2019 ▸) and (*E*)-2-[(2-hy­droxy-5-meth­oxy­benzyl­idene)amino]­benzo­nitrile (energy gap = 3.520 eV; Saraçoğlu *et al.*, 2020 ▸).

## Database survey   

A search of the Cambridge Structural Database (CSD, version 5.39, update of November 2019; Groom *et al.*, 2016 ▸) gave 14 hits for a 2-{[(2-hy­droxy-5-methyl­phen­yl)methyl­idene]amino}­benzo­nitrile moiety. The eight most closely related compounds are (*E*)-2-[(5-bromo-2-hy­droxy­benzyl­idene)amino]­benzo­nitrile (FOWXOF; Zhou *et al.*, 2009*a* ▸), 5-chloro-2-(2-hy­droxy­benzyl­idene­amino)­benzo­nitrile (GEJGAE; Cheng *et al.*, 2006 ▸), 2-{[(2-hy­droxy-5-meth­oxy­phen­yl)methyl­idene]amino}­benzo­nitrile (GOGYUZ; Faizi *et al.*, 2019 ▸), *trans*-2-(2-hy­droxy­benzyl­idene­amino)­benzo­nitrile (LOCBOV; Xia *et al.*, 2008 ▸), 2-[(2-hy­droxy-6-meth­oxy­benzyl­idene)amino]­benzo­nitrile (LOVDUX; Demircioğlu *et al.*, 2015 ▸), (*E*)-2-(2,4 di­hydroxy­benzyl­idene­amino)­benzo­nitrile (MOZPAT; Liu, 2009 ▸), (*E*)-2-(4-di­ethyl­amino-2-hy­droxy­benzyl­idene­amino)­ben­zo­nitrile (PUJDOO; Wang *et al.*, 2010 ▸) and (*E*)-2-[(3,5-di-*tert*-butyl-2-hy­droxy­benzyl­idene)amino]­benzo­nitrile (YOVBUH; Zhou *et al.*, 2009*b* ▸). All of these compounds are enol–imine tautomers, feature an *E* imine configuration and have the same common strong intra­molecular O—H⋯N hydrogen-bonding inter­action that stabilizes the mol­ecular conformation and forms an *S*(6) ring motif. The dihedral angles between the aromatic rings are generally smaller than the value of 25.65 (3)° observed for the title compound, with angles between 1.09 (4)° (for FOWXOF and GEJGAE) and 13.84 (13)° (for PUJDOO). Only YOVBUH features angles similar to those of (I), with dihedral angles of 21.74 (5), 27.59 (5) and 27.87 (5)° for the three independent mol­ecules in its structure. Steric crowding within each mol­ecule seem to be no issue for the eight structures analysed, and the varying torsion angles might be the result of subtle effects from crystal packing forces.

## Synthesis and crystallization   

The title compound was prepared by combining solutions of 2-hy­droxy-5-methyl-benzaldehyde (38.0 mg, 0.25 mmol) in ethanol (15 ml) and 2-amino­benzo­nitrile (33.0 mg, 0.25 mmol) in ethanol (15 ml) and stirring the mixture for 5 h under reflux (yield 60%, m.p. 412–414 K). Single crystals of the title compound suitable for X-ray analysis were obtained by slow evaporation of an ethanol solution.

## Refinement   

Crystal data, data collection and structure refinement details are summarized in Table 3 ▸. C-bound H atoms were positioned geometrically and refined using a riding model, with C—H = 0.93–0.97 Å and *U*_iso_(H) = 1.2–1.5*U*_eq_(C). The position of the H1 atom was obtained from a difference map; it was placed in a calculated position with a fixed C—O—H angle, but the O—H distance and the torsion angle were allowed to freely refine.

## Supplementary Material

Crystal structure: contains datablock(s) I. DOI: 10.1107/S2056989020008907/zl2787sup1.cif

Structure factors: contains datablock(s) I. DOI: 10.1107/S2056989020008907/zl2787Isup2.hkl

Supporting information file. DOI: 10.1107/S2056989020008907/zl2787Isup3.cml

CCDC reference: 2013269

Additional supporting information:  crystallographic information; 3D view; checkCIF report

## Figures and Tables

**Table 1:** Hydrogen-bond geometry (Å, °) *Cg*1 is the centroid of the C9–C14 ring.

*D*—H⋯*A*	*D*—H	H⋯*A*	*D*⋯*A*	*D*—H⋯*A*
O1—H1⋯N2	0.92	1.83	2.6280 (16)	145
C4—H4⋯N1^i^	0.93	2.77	3.610 (3)	150
C15—H15*A*⋯O1^ii^	0.96	2.74	3.641 (3)	158
C11—H11⋯*Cg*1^iii^	0.93	2.85	3.654 (16)	146

Symmetry codes: (i) 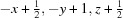; (ii) 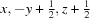; (iii) 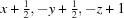.

**Table 2:** Comparison of selected observed (X-ray data) and calculated (DFT) geometric parameters (Å, °)

Parameter	X-ray	B3LYP/6–311G(d,p)
O1—C10	1.3503 (17)	1.3366
N2—C8	1.2795 (17)	1.2909
N2—C7	1.4130 (18)	1.3979
C1—N1	1.138 (2)	1.155
C1—C2	1.436 (3)	1.429
C8—C9	1.4380 (19)	1.4432
		
N1—C1—C2	179.2 (2)	178.3
C8—N2—C7	121.58 (13)	121.07
N2—C8—C9	122.03 (13)	122.79
		
C7—N2—C8—C9	−178.75 (13)	−176.57

**Table 3:** Experimental details

Crystal data	
Chemical formula	C_15_H_12_N_2_O
*M*_r_	236.27
Crystal system, space group	Orthorhombic, *P* *b* *c* *a*
Temperature (K)	296
*a*, *b*, *c* (Å)	7.8139 (3), 27.047 (1), 11.7683 (5)
*V* (Å^3^)	2487.14 (17)
*Z*	8
Radiation type	Mo *K*α
μ (mm^−1^)	0.08
Crystal size (mm)	0.73 × 0.42 × 0.24

Data collection	
Diffractometer	Stoe IPDS 2
Absorption correction	Integration (*X-RED32*; Stoe & Cie, 2002 ▸)
*T*_min_, *T*_max_	0.951, 0.989
No. of measured, independent and observed [*I* > 2σ(*I*)] reflections	15314, 2270, 1552
*R*_int_	0.040
(sin θ/λ)_max_ (Å^−1^)	0.602

Refinement	
*R*[*F* ^2^ > 2σ(*F* ^2^)], *wR*(*F* ^2^), *S*	0.038, 0.103, 1.04
No. of reflections	2270
No. of parameters	167
H-atom treatment	H atoms treated by a mixture of independent and constrained refinement
Δρ_max_, Δρ_min_ (e Å^−3^)	0.10, −0.09

Computer programs: *X-AREA* and *X-RED32* (Stoe & Cie, 2002 ▸), *SHELXT2018/3* (Sheldrick, 2015*a* ▸), *SHELXL2018/3* (Sheldrick, 2015*b* ▸), *OLEX2* (Dolomanov *et al.*, 2009 ▸), *Mercury* (Macrae *et al.*, 2020 ▸), *WinGX* (Farrugia, 2012 ▸), *PLATON* (Spek, 2020 ▸) and *publCIF* (Westrip, 2010 ▸).

## supplementary crystallographic information

### Crystal data

**Table:**

C_15_H_12_N_2_O	*D*_x_ = 1.262 Mg m^−^^3^
*M**_r_* = 236.27	Mo *K*α radiation, λ = 0.71073 Å
Orthorhombic, *P**b**c**a*	Cell parameters from 13160 reflections
*a* = 7.8139 (3) Å	θ = 1.5–25.8°
*b* = 27.047 (1) Å	µ = 0.08 mm^−^^1^
*c* = 11.7683 (5) Å	*T* = 296 K
*V* = 2487.14 (17) Å^3^	Prism, colorless
*Z* = 8	0.73 × 0.42 × 0.24 mm
*F*(000) = 992	

### Data collection

**Table:**

STOE IPDS 2 diffractometer	2270 independent reflections
Radiation source: sealed X-ray tube, 12 x 0.4 mm long-fine focus	1552 reflections with *I* > 2σ(*I*)
Plane graphite monochromator	*R*_int_ = 0.040
Detector resolution: 6.67 pixels mm^-1^	θ_max_ = 25.3°, θ_min_ = 1.5°
rotation method scans	*h* = −9→9
Absorption correction: integration (X-RED32; Stoe & Cie, 2002)	*k* = −32→32
*T*_min_ = 0.951, *T*_max_ = 0.989	*l* = −14→14
15314 measured reflections	

### Refinement

**Table:**

Refinement on *F*^2^	Secondary atom site location: difference Fourier map
Least-squares matrix: full	Hydrogen site location: inferred from neighbouring sites
*R*[*F*^2^ > 2σ(*F*^2^)] = 0.038	H atoms treated by a mixture of independent and constrained refinement
*wR*(*F*^2^) = 0.103	*w* = 1/[σ^2^(*F*_o_^2^) + (0.0529*P*)^2^ + 0.0539*P*] where *P* = (*F*_o_^2^ + 2*F*_c_^2^)/3
*S* = 1.04	(Δ/σ)_max_ < 0.001
2270 reflections	Δρ_max_ = 0.10 e Å^−^^3^
167 parameters	Δρ_min_ = −0.09 e Å^−^^3^
0 restraints	Extinction correction: SHELXL-2018/3 (Sheldrick 2018), Fc^*^=kFc[1+0.001xFc^2^λ^3^/sin(2θ)]^-1/4^
Primary atom site location: dual	Extinction coefficient: 0.0048 (9)

### Special details

**Table:**

Geometry. All esds (except the esd in the dihedral angle between two l.s. planes) are estimated using the full covariance matrix. The cell esds are taken into account individually in the estimation of esds in distances, angles and torsion angles; correlations between esds in cell parameters are only used when they are defined by crystal symmetry. An approximate (isotropic) treatment of cell esds is used for estimating esds involving l.s. planes.

### Fractional atomic coordinates and isotropic or equivalent isotropic displacement parameters (Å^2^)

**Table:**

	*x*	*y*	*z*	*U*_iso_*/*U*_eq_	
C1	0.4510 (2)	0.43624 (6)	0.56936 (18)	0.0817 (5)	
C2	0.4377 (2)	0.43849 (6)	0.69096 (14)	0.0740 (4)	
C3	0.3751 (3)	0.48102 (6)	0.7420 (2)	0.0926 (5)	
H3	0.343203	0.507974	0.697713	0.111*	
C4	0.3606 (3)	0.48315 (8)	0.8578 (2)	0.1034 (6)	
H4	0.316746	0.511334	0.892460	0.124*	
C5	0.4108 (3)	0.44357 (8)	0.92255 (18)	0.0991 (6)	
H5	0.401374	0.445315	1.001220	0.119*	
C6	0.4752 (2)	0.40119 (6)	0.87308 (15)	0.0836 (5)	
H6	0.510544	0.374922	0.918342	0.100*	
C7	0.48704 (18)	0.39788 (5)	0.75604 (13)	0.0686 (4)	
C8	0.54747 (18)	0.31307 (5)	0.73859 (12)	0.0641 (4)	
H8	0.499419	0.308529	0.810127	0.077*	
C9	0.61539 (17)	0.27095 (5)	0.67918 (11)	0.0598 (4)	
C10	0.69055 (18)	0.27512 (6)	0.57110 (12)	0.0654 (4)	
C11	0.7545 (2)	0.23368 (6)	0.51800 (14)	0.0759 (4)	
H11	0.805539	0.236438	0.446900	0.091*	
C12	0.7430 (2)	0.18837 (7)	0.56968 (15)	0.0800 (5)	
H12	0.787489	0.160829	0.532733	0.096*	
C13	0.6667 (2)	0.18226 (6)	0.67585 (15)	0.0780 (5)	
C14	0.6052 (2)	0.22403 (5)	0.72774 (13)	0.0696 (4)	
H14	0.554152	0.220906	0.798775	0.084*	
C15	0.6544 (3)	0.13194 (6)	0.7305 (2)	0.1221 (8)	
H15A	0.673066	0.134971	0.810847	0.183*	
H15B	0.739587	0.110501	0.698457	0.183*	
H15C	0.542781	0.118329	0.717044	0.183*	
O1	0.70348 (16)	0.31893 (4)	0.51676 (10)	0.0877 (4)	
H1	0.654 (3)	0.3432 (6)	0.5601 (13)	0.132*	
N1	0.4603 (3)	0.43494 (6)	0.47293 (15)	0.1085 (6)	
N2	0.55113 (16)	0.35659 (5)	0.69604 (10)	0.0681 (3)	

### Atomic displacement parameters (Å^2^)

**Table:**

	*U*^11^	*U*^22^	*U*^33^	*U*^12^	*U*^13^	*U*^23^
C1	0.0981 (12)	0.0574 (9)	0.0896 (13)	0.0001 (8)	−0.0131 (11)	−0.0016 (9)
C2	0.0722 (9)	0.0636 (9)	0.0861 (11)	−0.0043 (7)	−0.0017 (8)	−0.0097 (9)
C3	0.0903 (12)	0.0715 (11)	0.1160 (15)	0.0009 (9)	−0.0017 (11)	−0.0165 (11)
C4	0.1000 (14)	0.0856 (13)	0.1245 (18)	0.0013 (11)	0.0128 (13)	−0.0354 (13)
C5	0.1078 (14)	0.0977 (14)	0.0919 (13)	−0.0108 (12)	0.0155 (11)	−0.0295 (12)
C6	0.0909 (12)	0.0832 (11)	0.0766 (11)	−0.0075 (9)	0.0047 (9)	−0.0145 (9)
C7	0.0653 (9)	0.0666 (9)	0.0738 (10)	−0.0066 (7)	0.0033 (7)	−0.0123 (8)
C8	0.0613 (8)	0.0729 (9)	0.0582 (8)	−0.0055 (7)	0.0024 (7)	−0.0020 (7)
C9	0.0557 (7)	0.0681 (9)	0.0558 (8)	−0.0041 (6)	−0.0009 (6)	−0.0045 (7)
C10	0.0618 (8)	0.0749 (10)	0.0597 (8)	−0.0040 (7)	−0.0004 (7)	−0.0017 (8)
C11	0.0704 (9)	0.0948 (12)	0.0624 (9)	0.0027 (9)	0.0066 (7)	−0.0124 (9)
C12	0.0785 (10)	0.0809 (11)	0.0804 (11)	0.0103 (8)	0.0002 (9)	−0.0180 (9)
C13	0.0823 (11)	0.0691 (10)	0.0827 (11)	0.0009 (8)	0.0020 (9)	−0.0063 (8)
C14	0.0729 (9)	0.0715 (9)	0.0645 (9)	−0.0027 (7)	0.0048 (7)	−0.0020 (8)
C15	0.156 (2)	0.0725 (12)	0.1380 (18)	0.0078 (13)	0.0243 (15)	0.0080 (12)
O1	0.1075 (9)	0.0844 (8)	0.0713 (7)	−0.0015 (7)	0.0194 (6)	0.0055 (6)
N1	0.1567 (16)	0.0760 (10)	0.0927 (12)	0.0039 (9)	−0.0181 (11)	0.0018 (9)
N2	0.0721 (8)	0.0658 (8)	0.0663 (7)	−0.0029 (6)	0.0019 (6)	−0.0029 (6)

### Geometric parameters (Å, º)

**Table:**

C1—N1	1.138 (2)	C9—C14	1.3940 (19)
C1—C2	1.436 (3)	C9—C10	1.406 (2)
C2—C3	1.387 (2)	C10—O1	1.3503 (17)
C2—C7	1.393 (2)	C10—C11	1.377 (2)
C3—C4	1.369 (3)	C11—C12	1.371 (2)
C3—H3	0.9300	C11—H11	0.9300
C4—C5	1.371 (3)	C12—C13	1.394 (2)
C4—H4	0.9300	C12—H12	0.9300
C5—C6	1.381 (2)	C13—C14	1.371 (2)
C5—H5	0.9300	C13—C15	1.509 (2)
C6—C7	1.383 (2)	C14—H14	0.9300
C6—H6	0.9300	C15—H15A	0.9600
C7—N2	1.4130 (18)	C15—H15B	0.9600
C8—N2	1.2795 (17)	C15—H15C	0.9600
C8—C9	1.4380 (19)	O1—H1	0.92 (2)
C8—H8	0.9300		
			
N1—C1—C2	179.2 (2)	C10—C9—C8	122.05 (13)
C3—C2—C7	120.90 (16)	O1—C10—C11	118.18 (14)
C3—C2—C1	119.48 (16)	O1—C10—C9	122.02 (14)
C7—C2—C1	119.62 (14)	C11—C10—C9	119.80 (15)
C4—C3—C2	119.7 (2)	C12—C11—C10	120.16 (15)
C4—C3—H3	120.2	C12—C11—H11	119.9
C2—C3—H3	120.2	C10—C11—H11	119.9
C3—C4—C5	119.78 (19)	C11—C12—C13	122.10 (15)
C3—C4—H4	120.1	C11—C12—H12	119.0
C5—C4—H4	120.1	C13—C12—H12	119.0
C4—C5—C6	121.21 (19)	C14—C13—C12	116.84 (15)
C4—C5—H5	119.4	C14—C13—C15	122.08 (17)
C6—C5—H5	119.4	C12—C13—C15	121.08 (16)
C5—C6—C7	119.87 (18)	C13—C14—C9	123.19 (15)
C5—C6—H6	120.1	C13—C14—H14	118.4
C7—C6—H6	120.1	C9—C14—H14	118.4
C6—C7—C2	118.54 (14)	C13—C15—H15A	109.5
C6—C7—N2	124.92 (15)	C13—C15—H15B	109.5
C2—C7—N2	116.51 (14)	H15A—C15—H15B	109.5
N2—C8—C9	122.03 (13)	C13—C15—H15C	109.5
N2—C8—H8	119.0	H15A—C15—H15C	109.5
C9—C8—H8	119.0	H15B—C15—H15C	109.5
C14—C9—C10	117.90 (14)	C10—O1—H1	109.5
C14—C9—C8	120.05 (13)	C8—N2—C7	121.58 (13)
			
C7—C2—C3—C4	−0.4 (3)	C14—C9—C10—C11	−1.4 (2)
C1—C2—C3—C4	179.34 (17)	C8—C9—C10—C11	179.69 (14)
C2—C3—C4—C5	1.2 (3)	O1—C10—C11—C12	−179.53 (15)
C3—C4—C5—C6	−0.4 (3)	C9—C10—C11—C12	0.8 (2)
C4—C5—C6—C7	−1.1 (3)	C10—C11—C12—C13	0.4 (3)
C5—C6—C7—C2	1.9 (2)	C11—C12—C13—C14	−0.9 (3)
C5—C6—C7—N2	179.77 (15)	C11—C12—C13—C15	179.52 (18)
C3—C2—C7—C6	−1.1 (2)	C12—C13—C14—C9	0.3 (2)
C1—C2—C7—C6	179.13 (15)	C15—C13—C14—C9	179.81 (17)
C3—C2—C7—N2	−179.21 (14)	C10—C9—C14—C13	0.9 (2)
C1—C2—C7—N2	1.1 (2)	C8—C9—C14—C13	179.80 (14)
N2—C8—C9—C14	−178.55 (13)	C9—C8—N2—C7	−178.75 (13)
N2—C8—C9—C10	0.3 (2)	C6—C7—N2—C8	25.3 (2)
C14—C9—C10—O1	178.93 (14)	C2—C7—N2—C8	−156.72 (13)
C8—C9—C10—O1	0.0 (2)		

### Hydrogen-bond geometry (Å, º)

*Cg*1 is the centroid of the C9–C14 ring.

**Table:**

*D*—H···*A*	*D*—H	H···*A*	*D*···*A*	*D*—H···*A*
O1—H1···N2	0.92	1.83	2.6280 (16)	145
C4—H4···N1^i^	0.93	2.77	3.610 (3)	150
C15—H15*A*···O1^ii^	0.96	2.74	3.641 (3)	158
C11—H11···*Cg*1^iii^	0.93	2.85	3.654 (16)	146

Symmetry codes: (i) −*x*+1/2, −*y*+1, *z*+1/2; (ii) *x*, −*y*+1/2, *z*+1/2; (iii) *x*+1/2, −*y*+1/2, −*z*+1.
